# Adherence to multiple recommendations of the Dietary Guidelines for the Brazilian Population (*Guia Alimentar para a População Brasileira*) and associated factors among adolescents: a cross-sectional study with data from the National Students’ Health Survey, 2019

**DOI:** 10.1590/S2237-96222025v34e20240404.en

**Published:** 2025-06-13

**Authors:** Diôgo Vale, Maria Eduarda da Costa Andrade, Natalie Marinho Dantas

**Affiliations:** 1 Instituto Federal de Educação, Ciência e Tecnologia do Rio Grande do Norte, Ceará-Mirim, RN, Brazil; 2 Instituto Federal de Educação, Ciência e Tecnologia do Rio Grande do Norte, Natal, RN, Brazil; 3 Universidade Estadual de Campinas, Faculdade de Engenharia de Alimentos, Departamento de Ciência de Alimentos e Nutrição, Campinas, SP, Brazil

**Keywords:** Adolescent, Dietary Guidelines, Diet, Social Factors, Cross-Sectional Studies, Adolescente, Orientaciones Dietéticas, Dieta, Factores Sociales, Estudios Transversales

## Abstract

**Objective:**

To analyze the prevalence of adherence to multiple recommendations of the Dietary Guidelines for the Brazilian Population among school adolescents in Brazil in 2019 and its associations with sociodemographic and behavioral factors.

**Methods:**

Cross-sectional study with micro data from the 2019 National Students’ Health Survey. The dependent variable was adherence to multiple Guidelines recommendations, based on dietary practices markers. The independent variables were sex, race/skin color, maternal education, area of ​​residence, geographic region, school administration, territory of residence, use of laxative measures, formulas for weight loss or weight gain, body satisfaction and self-perception of health. Prevalence ratios (PR) and 95% confidence intervals (95%CI) were calculated by Poisson regression.

**Results:**

The prevalence of better adherence to multiple Guidelines recommendations among Brazilian adolescents was 0.4% (95%CI 0.3; 0.4). This adherence to the recommendations showed a higher rate with factors such as: living in rural areas (PR 1.91; 95%CI 1.16; 3.13), in the Northeast region (PR 1.92; 95%CI 1.10; 3.35 and the Midwest region (PR 2.29; 95%CI 1.28; 4.09), in areas outside the capitals (PR 1.33; 95%CI 1.01; 1.77), declaring oneself very satisfied with one’s body (PR 2.72; 95%CI 1.20; 6.20) and considering oneself as having a very good health (PR 3.87; 95%CI 1.12; 13.43) or poor health (PR 6.11; 95%CI 1.20; 31.12).

**Conclusion:**

The prevalence of adherence to multiple Guidelines recommendations among adolescents is very low and the factors associated with better adherence are characterized by less urbanized areas and, above all, with positive perceptions about one’s body and health.

## Resumen

**Objetivo:**

Analizar la prevalencia de la adhesión a diversas recomendaciones de la *Guía Alimentaria para la Población Brasileña* entre adolescentes escolares en Brasil en 2019 y sus asociaciones con factores sociodemográficos y de comportamentales.

**Métodos:**

Estudio transversal con microdatos de la Encuesta Nacional de Salud Estudiantil 2019. La variable dependiente fue la adhesión a diversas recomendaciones de Guidelines, basadas en marcadores de prácticas dietéticas. Las variables independientes fueron sexo, raza/color de piel, educación materna, área de residencia, región geográfica, administración escolar, territorio de residencia, uso de medidas laxantes, fórmulas para la pérdida o aumento de peso, satisfacción corporal y autopercepción de la salud. Las razones de prevalencia (PR) y los intervalos de confianza del 95% (IC del 95%) se calcularon mediante regresión de Poisson.

**Resultados:**

La prevalencia de una mejor adhesión a múltiples recomendaciones de la Guidelines entre los adolescentes brasileños fue del 0,4% (IC95% 0,3; 0,4). Esta adhesión a las recomendaciones mostró una tasa más alta con factores como: vivir en áreas rurales (PR 1,91; IC95% 1,16; 3.13), en la región Noreste (PR 1,92; IC95% 1,10; 3,35) y en la región Medio Oeste (PR 2,29; IC95% 1,28; 4,09), en áreas fuera de las capitales (PR 1,33; CI95% 1,01; 1,77), declarándose muy satisfecho con el propio cuerpo (PR 2,72; 95% CI 1,20; 6,20) y considerándose con muy buena salud (PR 3,87; CI95% 1,12; 13,43) o mala salud (PR 6,11; CI95% 1,20; 31,12).

**Conclusión:**

La prevalencia de la adhesión a diversas recomendaciones de la Guidelines entre los adolescentes es muy baja y los factores asociados con una mejor adhesión se caracterizan por áreas menos urbanizadas y, sobre todo, con percepciones positivas sobre el propio cuerpo y la salud.

**Ethical aspects**

This research complies with ethical principles and the micro database of the National Students’ Health Survey (PeNSE 2019) made available by IBGE was used for the analyses. PeNSE 2019 was approved by Conep Opinion No. 3.249.268, dated April 8, 2019.:

## Introduction

Inadequate nutrition and overweight in adolescence represent a challenge for Brazilian public health, significantly increasing the incidence and prevalence of chronic non-communicable diseases. Recent studies show a worrying increase in the prevalence of overweight and obesity in this population, making it urgent to implement effective measures to reverse this scenario (1,2).

Excessive consumption of ultra-processed foods (27% of energy in 2017-2018) and low consumption of unprocessed or minimally processed food, such as fruits, greens and vegetables in the diet of Brazilian adolescents (3,4) are a complex issue, influenced by social and emotional factors (5,6).

Faced with these problems, the Brazilian Ministry of Health has been working to promote healthy eating and combat the rise in overweight and obesity. With this as our goal, the Strategic Action Plan to Prevent Chronic Diseases and Non-Communicable Diseases in Brazil 2021-2030 (7) and the Dietary Guidelines for the Brazilian Population (8) were published. The latter, with clear guidelines, served as a basis for initiatives such as the Protocol for the use of Guidelines in adolescence for nutritional counseling in Primary Care (9) and Resolution 6/2020 of the National School Feeding Program (PNAE), which sought to strengthen healthy eating for this public in the school setting (10).

Despite government initiatives, such as the publication of the Guidelines (8), which emphasizes healthy and sustainable eating practices, there is still a significant gap in knowledge about Brazilian adolescents’ adherence to its recommendations. Guidelines addresses not only food choices, but the sociocultural aspects of eating, such as eating mindfully and with others. However, the influence of these sociocultural factors on adolescents’ eating practices and how they apply the Guidelines recommendations are yet to be more studied (11).

The consumption of ultra-processed foods by adolescents is known to vary according to sociodemographic factors (12,13). However, additional research is needed, considering social and behavioral aspects, such as body dissatisfaction and risk behaviors for eating disorders, which are associated with greater or lesser adherence to Guidelines recommendations.

Therefore, this study aims to fill this gap, analyzing the prevalence of adherence to multiple Guidelines recommendations among school adolescents in Brazil in 2019 and its associations with sociodemographic and behavioral factors. The results of this research will contribute to the assessment of the potential and challenges in using this guide to promote adequate and healthy eating among this age group.

## Methods

### Study design and data source

This was a cross-sectional/observational study conducted as a school-based survey (individualized data). The data source for this quantitative, descriptive and analytical research was the 2019 National Students’ Health Survey (PeNSE 2019). This was the fourth edition of this school-based research developed by the Brazilian Institute of Geography and Statistics. PeNSE consists of a school-based survey that collects information on the health of Brazilian adolescents and is essential for food and nutritional surveillance of this age group (14).

### Context, participants and study size

The PeNSE 2019 sample was cluster-based, probabilistic and comprised students aged 13 to 17, enrolled and regularly attending public and private schools throughout the country. The selection of schools followed a two-stage design, with stratification by region and school size. Data were collected from 4,242 schools and 6,612 classes, resulting in a final sample of 159,245 adolescents with valid questionnaires (14).

Data collection was carried out between April 9 and September 30, 2019 through a self-administered questionnaire with adolescent students in the classroom. The questionnaire covered topics related to adolescent health, including eating habits, physical activity, mental health, sexual and reproductive health, among others (14).

PeNSE was approved by the National Research Ethics Committee under registration No. 3,249,268 on April 8, 2019 (14). This work complies with the ethical precepts set out in Resolution No. 510, of April 7, 2016, of the National Health Council (15).

The results presented in this study relate to 153,153 adolescents, which corresponds to 96.2% of the initial sample of PENSE 2019. To ensure consistency in the analyses, 3.8% of the individuals in the initial sample were categorized as “absent” (99), when there was missing data for any of the dietary practice variables that originated the ‘dependent variable’. This approach was used to maintain the weights of the original database, ensuring that the adequate effect of the sampling design was preserved.

### Variables

The dependent variable was adherence to multiple Guidelines recommendations. This variable was developed by the study authors based on the 22 markers of eating practices available in the PeNSE 2019 micro database. Initially, these markers were distributed according to Guidelines’s “ten steps for an adequate and healthy diet” (8). During this process, we identified markers that characterized in a straightforward way only four steps of the Guide: (a) making natural or minimally processed food the basis of the diet; (b) avoiding the consumption of ultra-processed foods; (c) eating regularly and attentively, in appropriate environments and, whenever possible, with other people; and (d) when away from home, giving preference to places that serve freshly prepared meals (Table 1).

**Table 1:** Markers of eating habits from the 2019 National Students’ Health Survey according to recommendations from the Dietary Guidelines for the Brazilian Population for the development of variables of adherence to multiple Guidelines recommendations among adolescents. Brazil, 2019

Recommendations from the Dietary Guidelines for the Brazilian Population with direct markers available in the National School Health Survey	Multiple adherence to Guidelines recommendations with categories of ideal recommended dietary practices (more restricted)	Multiple adherence to Guidelines recommendations with “possible” recommended dietary practices categories (less restricted)
**1 Make unprocessed or minimally processed foods the basis of your diet**		
1.1 In the last 7 days, on how many days did you eat beans?	0-6 days/week [0] 7 days/week [1]	0-6 days/week [0] 7 days/week [1]
1.2 In the last 7 days, on how many days did you eat fresh fruit or fruit salad?	0-6 days/week [0] 7 days/week [1]	0-6 days/week [0] 7 days/week [1]
1.3 In the last 7 days, on how many days did you eat at least one type of vegetable other than potatoes or cassava?	0-6 days/week [0] 7 days/week [1]	0-6 days/week [0] 7 days/week [1]
**2 Avoid consuming ultra-processed foods**		
2.1 In the last 7 days, on how many days did you drink soda?	1-7 days/week [0] 0 days/week [1]	3-7 days/week (0) 2 days or less/week (1)
2.2 In the last 7 days, on how many days did you eat sweet treats, such as candies, sweets, chocolate, chewing gum, lollipops and others?	1-7 days/week [0] 0 days/week [1]	3-7 days/week (0) 2 days or less/week (1)
2.3 Sum of consumption of ultra-processed foods on the day before the survey (2.3.1 to 2.3.13)	Consumed 1-13 foods [0] Did not consume any food [1]	Consumed 4-13 foods [0] Consumed 3 or fewer items in the previous day - 25th percentile [1]
2.3.1 Did you drink soda yesterday?	No [0] Yes [1]	No [0] Yes [1]
2.3.2 Yesterday, did you drink fruit juice from a carton or can?	No [0] Yes [1]	No [0] Yes [1]
2.3.3 Did you drink powdered drink mixes yesterday?	No [0] Yes [1]	No [0] Yes [1]
2.3.4 Did you drink chocolate milk yesterday?	No [0] Yes [1]	No [0] Yes [1]
2.3.5 Did you have flavored yogurt yesterday?	No [0] Yes [1]	No [0] Yes [1]
2.3.6 Yesterday, did you eat packaged snacks (chips) or crackers/salty biscuits?	No [0] Yes [1]	No [0] Yes [1]
2.3.7 Yesterday, did you eat cookies or sweet biscuits, stuffed cookies or packaged cupcakes?	No [0] Yes [1]	No [0] Yes [1]
2.3.8 Yesterday, did you eat chocolate, ice cream, gelatin, flan or other industrialized dessert?	No [0] Yes [1]	No [0] Yes [1]
2.3.9 Yesterday, did you eat sausage, salami, mortadella or ham?	No [0] Yes [1]	No [0] Yes [1]
2.3.10 Yesterday, did you eat sliced bread, hot dog buns or hamburger buns?	No [0] Yes [1]	No [0] Yes [1]
2.3.11 Did you eat margarine yesterday?	No [0] Yes [1]	No [0] Yes [1]
2.3.12 Yesterday, did you eat mayonnaise, ketchup or other industrial sauces?	No [0] Yes [1]	No [0] Yes [1]
2.3.13 Yesterday, did you eat instant noodles, packaged soup, frozen lasagna or another ready-made frozen dish?	No [0] Yes [1]	No [0] Yes [1]
**3 Eat regularly and attentively, in appropriate environments and, whenever possible, with others**		
3.1 Do you usually have breakfast?	0-6 days/week [0] 7 days/week [1]	0-6 days/week (0) 7 days/week (1)
3.2 Do you usually have lunch or dinner with your mother, father or guardian?	0-6 days/week [0] 7 days/week [1]	0-6 days/week (0) 7 days/week (1)
3.3 During your meals, how often do you eat while doing something else (watching TV, using the computer or cell phone)?	1 or more days/week [0] Never [1]	3 or more days/week [0] 2 days or less [1]
**4 When away from home, give preference to places that serve freshly prepared meals**		
4.1 How many days of the last week did you eat at snack bars, hot dog stands, pizzerias, fast food restaurants, etc.?	1 or more days/week [0] 0 days/week [1]	3 or more days/week [0] 2 days or less [1]
**Recommendations from the Dietary Guidelines for the Brazilian Population without direct markers available in the National Students’ Health Survey**		
Use oil, fats, salt and sugar in small quantities when seasoning and cooking food and creating culinary preparations		
Limit consumption of processed foods		
Shop at places that offer a variety of unprocessed or minimally processed foods		
Develop, exercise and share culinary skills		
Plan your time to give food the space it deserves		
Be critical of information, guidance and messages about food conveyed in commercial ads		

^a^Numbers in brackets represent point values assigned to each category for the calculation of the two multiple adherence models.

Subsequently, each dietary practice marker had its responses categorized as “0” (did not meet the Guidelines recommendation) and “1” (met the Guidelines recommendation). The assessment of adherence to multiple Guidelines recommendations involved the use of the total score resulting from the sum of the markers: 1.1, 1.2, 1.3, 2.1, 2.2, 2.3, 3.1, 3.2, 3.3 and 4.1. Therefore, this dependent variable assumed values from “0” to “10” (Table 1).

Considering the subsequent steps of the statistical analysis, this main variable was calculated in two ways: (a) multiple adherence to Guidelines recommendations, with categories of “ideal recommended” dietary practices (more restrictive), characterized, for example, by the consumption of unprocessed foods and/or minimally processed foods on all days of the week and absence of ultra-processed foods consumption; and (b) multiple adherence to Guidelines recommendations with categories of “possible recommended” (less restrictive) dietary practices, with ultra-processed foods consumption, for example, tolerated up to twice a week. The two ways of calculating this variable was developed due to the impossibility of carrying out modeling with the most restricted category (a) (Table 2).

**Table 2:** Prevalence (%) and confidence intervals (95%CI) of multiple adherence to multiple recommendations of the Dietary Guide for the Brazilian Population (Guidelines) according to ideal (more restricted) and possible (less restricted) recommended categories among school adolescents. Brazil, 2019 (n=153,153^a^)

Number of dietary practices adhering to Guidelines	Adherence to Guidelines recommendations with categories of ideal recommended dietary practices (more restricted)		Adherence to Guidelines recommendations with possible recommended dietary practices categories (less restricted)	
	%	95%CI	%	95%CI
0	4.6	4.4; 4.8	1.1	0.9; 1.2
1	11.8	11.5; 12.2	4.1	3.9; 4.4
2	18.8	18.4; 19.3	9.3	9.0; 9.6
3	21.4	21.0; 21.9	15.4	15.1; 15.8
4	18.9	18.5; 19.3	19.5	19.1; 19.9
5	13.3	12.9; 13.6	19.3	18.8; 19.7
6	6.8	6.5; 7.0	15.5	15.1; 15.9
7	3.0	2.8; 3.2	9.6	9.3; 9.9
8	1.1	1.0; 1.2	4.4	4.2; 4.6
9	0.2	0.2; 0.3	1.5	1.4; 1.6
10 (highest adherence)	0.0	0.0; 0.1	0.4	0.3; 0.4

^a^Number of adolescents with data collected and valid for this study. When considering the sample effect, the data represent 15,192,972 Brazilian adolescents.

The creation of these models a priori was based on the qualitative recommendations of the Guidelines, which does not present references on quantities and frequency of dietary practices, but recommends the incorporation of these nutrition and health-promoting practices, together with a healthier lifestyle. (8)

Two dimensions of independent variables were considered in this study:

- sociodemographic factors - sex (male and female), self-declared race/skin color (white, black, brown, yellow for Asians and indigenous), maternal education (Did not study, Incomplete elementary school, Complete elementary school, Incomplete high school, Complete high school, Incomplete higher education, Complete higher education), area of residence (urban and rural), geographic region (North, Northeast, Southeast, South and Midwest), school administration (private and public), and territory of residence (capital, far away from the capital);
- behavioral factors - use of laxative measures (yes, no), use of weight loss formulas without professional supervision (yes, no), use of formulas for weight gain or muscle mass gain without professional supervision (yes, no), body satisfaction (very unhappy, unhappy, indifferent, happy, very happy), and self-perception of health (very good, good, fair, bad, very bad).

### Bias

To minimize possible biases, several methodological measures were used in this study. PeNSE 2019 sampling estimators were used to adjust the results and ensure the representativeness of the sample. The Guidelines adherence variable proposed by the authors was developed in a systematic and rigorous manner, based on the guide recommendations and the variables available in the research. Furthermore, the variables categorization was reviewed by three researchers in the field to ensure the consistency and reliability of data.

### Statistical methods

The prevalences and respective 95% confidence intervals (95% CI) were estimated for (a) multiple adherence to Guidelines recommendations with categories of ideal recommended dietary practices (more restrictive) and for (b) multiple adherence to Guidelines recommendations with categories of possible recommended dietary practices (less restrictive).

For bivariate and multivariate Poisson regressions, the dichotomous categorical variable of adherence to multiple Guidelines recommendations was used. This was developed taking into consideration the (b) multiple adherence to Guidelines recommendations with categories of possible recommended dietary practices (less restrictive). With these results, those adolescents who scored from zero to nine were in the reference category (some adherence to Guidelines with a score of “zero”) and those who scored for all ten practices were categorized as having greater adherence to Guidelines with a score of “one”. The decision to categorize “0” as “some adherence” was made because, even though the adolescents did not reach the “possible” model a priori, they had some positive eating practices incorporated into their routine according to the data collected. It is known that such positive actions also need to be valued in population groups, as the Guidelines does not determine weekly frequencies in its publication (8). 

Bivariate and multivariate Poisson regressions were applied to assess the associations of the dependent variable with the independent variables (sociodemographic and behavioral factors) based on estimates of Prevalence Ratios (PR) and respective 95%CI. The Backward method was used to create the multivariate regression model, where the variables related to a level of statistical significance less than 20% in the bivariate analysis were all included and, subsequently, those that did not reach the determining level of significance were removed one by one. Only those variables that presented p-value<0.05 remained in the final (multivariate) model. These analyses were conducted using Stata software, version 14.2, with the Survey module considering the estimators of the PeNSE 2019 sampling design.

## Results

Among the adolescents evaluated, the majority were female (51.4%), declared themselves to be white (36.0%) and lived in the Southeast region (40.2%) of Brazil.

Among Brazilian adolescent students, no one (0.0%; 95%CI 0.0; 0.1) managed to achieve the ten dietary practices evaluated to characterize multiple adherence to Guidelines recommendations with ideal categories (more restricted). When estimating multiple adherence to Guidelines recommendations with “possible” recommended dietary practice categories (less restricted), 0.4% (95% CI 0.3; 0.4) of the adolescents showed greater adherence to multiple Guidelines recommendations (Table 2).

Regarding the associated sociodemographic and behavioral factors that increased the prevalence of greater adherence to multiple Guidelines recommendations among adolescents, the following were included in the multivariate Poisson model: living in rural areas (PR 1.91; 95%CI 1.16; 3.13), living in the Northeast region (PR 1.92; 95%CI 1.10; 3.35) and living in the Midwest (PR 2.29; 95%CI 1.28; 4.09), living in areas outside the capitals (PR 1.33; 95%CI 1.01; 1.77), declaring oneself very happy with one’s body (PR 2.72; 95%CI 1.20; 6.20) and considering one’s own health as poor (PR 6.11; 95%CI 1.20; 31.12) or very good (PR 3.87; 95%CI 1.12; 13.43) (Table 3).

**Table 3:** Prevalence (%), Prevalence Ratios (PR) and confidence intervals (95%CI) of greater or some adherence to multiple recommendations for categories of possible recommended dietary practices (less restricted) of the Dietary Guidelines for the Brazilian Population among school adolescents according to sociodemographic and behavioral factors. Brazil, 2019 (n=153,153^a^)

Variable	Greater adherence to multiple Guidelines recommendations % (95%CI)	Some adherence to multiple Guidelines recommendations % (95%CI)	p-value	Bivariate model		Multivariate model
				PR (95%CI)	p-value	PR (95%CI) p-value
Sex			0.906			
Male	0.4 (0.3; 0.4)	99.6 (99.6; 99.7)				
Female	0.4 (0.3; 0.5)	99.6 (99.5; 99.7)				
**Area of residence**			0.001		<0.001	
Urban	0.3 (0.3; 0.4)	99.7 (99.6; 99.7)		Ref		Ref
Rural	0.8 (0.5; 1.2)	99.2 (98.8; 99.5)		2.50 (1.55; 4.04)		1.91 (1.16; 3.13) 0.011
**Race/skin color**			0.742			
White	0.4 (0.3; 0.5)	99.6 (99.5; 99.7)				
Black	0.3 (0.2; 0.5)	99.7 (99.5; 99.8)				
Yellow (Asian)	0.4 (0.2; 0.8)	99.6 (99.2; 99.8)				
Brown	0.4 (0.3; 0.5)	99.6 (99.5; 99.7)				
Indigenous	0.2 (0.1; 0.7)	99.8 (99.3; 99.9)				
**Maternal education**			0.254			
Uneducated	0.6 (0.2; 1.3)	99.4 (98.7; 99.8)				
Incomplete elementary education	0.3 (0.2; 0.5)	99.7 (99.5; 99.8)				
Completed elementary education	0.5 (0.3; 1.0)	99.5 (99.0; 99.7)				
Incomplete high school	0.3 (0.1; 0.5)	99.7 (99.5; 99.9)				
Completed high school	0.2 (0.1; 0.4)	99.8 (99.6; 99.9)				
Incomplete higher education	0.3 (0.1; 0.6)	99.7 (99.4; 99.9)				
Completed higher education	0.4 (0.3; 0.6)	99.6 (99.4; 99.7)				
**Geographic Region**			0.026			
South	0.2 (0.1; 0.3)	99.8 (99.7; 99.9)		Ref		Ref
Southeast	0.3 (0.2; 0.5)	99.7 (99.5; 99.8)		1.50 (0.74; 3.02)	0.260	1.53 (0.78; 3.02) 0.216
North	0.3 (0.2; 0.5)	99.7 (99.5; 99.8)		1.46 (0.77; 2.77)	0.244	1.16 (0.59; 2.89) 0.048
Midwest	0.5 (0.3; 0.6)	99.5 (99.4; 99.7)		2.27 (1.24; 4.14)	0.008	2.29 (1.28; 4.09) 0.005
North East	0.5 (0.4; 0.6)	99.5 (99.4; 99.6)		2.25 (1.24; 4.06)	0.007	1.92 (1.10; 3.35) 0.021
**School administration**			0.066			
Private	0.3 (0.2; 0.3)	99.7 (99.7; 99.8)		Ref		
Public	0.4 (0.3; 0.4)	99.6 (99.6; 99.7)		1.30 (0.92; 1.82)	0.137	
**Area of residence**			0.008			
Capital	0.2 (0.2; 0.3)	99.8 (99.7; 99.8)		Ref		Ref
Out of capital	0.4 (0.3; 0.5)	99.6 (99.5; 99.7)		1.54 (1.15; 1.06)	0.004	1.33 (1.01; 1.77) 0.048
**Laxative measures**			0.045			
No	0.4 (0.3; 0.4)	99.6 (99.6; 99.7)		Ref		
Yes	0.2 (0.1; 0.4)	99.8 (99.6; 99.9)		0.48 (0.23; 0.99)	0.048	
**Weight Loss Formula**			0.556			
No	0.3 (0.3; 0.4)	99.7 (99.6; 99.7)				
Yes	0.5 (0.2; 1.2)	99.5 (98.8; 99.8)				
**Weight Gain Formula**			0.126			
No	0.4 (0.3; 0.4)	99.6 (99.6; 99.7)		Ref		
Yes	0.2 (0.1; 0.4)	99.8 (99.6; 99.9)		0.49 (0.19; 1.26)	0.140	
**Body satisfaction**			<0.001			
Very unhappy	0.2 (0.1; 0.3)	99.8 (99.7; 99.9)		Ref		Ref
Unhappy	0.1 (0.1; 0.2)	99.9 (99.8; 99.9)		0.56 (0.25; 1.25)	0.154	0.61 (0.27; 1.37) 0.232
Indifferent	0.1 (0.1; 0.3)	99.9 (99.7; 99.9)		0.82 (0.42; 1.59)	0.558	0.92 (0.46; 1.85) 0.820
Happy	0.3 (0.2; 0.4)	99.7 (99.6; 99.8)		1.72 (0.79; 3.77)	0.174	1.76 (0.79; 3.93) 0.168
Very happy	0.7 (0.5; 0.8)	99.3 (99.2; 99.5)		3.71 (1.80; 7.68)	<0.001	2.72 (1.20; 6.20) 0.017
**Self-perception of general health status**			<0.001			
Very bad	0.2 (0.1; 0.4)	99.8 (99.6; 99.9)		Ref		Ref
Bad	0.6 (0.2; 1.6)	99.4 (98.4; 99.8)		3.61 (0.83; 15.68)	0.087	6.11 (1.20; 31.12) 0.029
Regular	0.2 (0.1; 0.2)	99.8 (99.8; 99.9)		0.99 (0.31; 3.15)	0.991	1.45 (0.38; 5.46) 0.585
Good	0.2 (0.1; 0.3)	99.8 (99.7; 99.9)		1.35 (0.44; 4.10)	0.595	1.68 (0.47; 6.05) 0.427
Very good	0.6 (0.5; 0.8)	99.4 (99.2; 99.5)		4.15 (1.41; 12.26)	0.010	3.87 (1.12; 13.43) 0.033

^a^Number of adolescents with data collected and valid for this study. When considering the sample effect, the data represent 15,192,972 Brazilian adolescents.

In practice, the values of these PR in the multivariate model represent increases in the outcome prevalence in relation to the sociodemographic and behavioral factors that remained associated. The prevalence of greater Guidelines adherence among adolescents was 91% higher among those living in rural areas compared to the prevalence of this outcome in urban areas. The prevalence of this type of adherence to Guidelines was also 92% higher in the Northeast region, and 129% in the Midwest, taking the prevalence in the North as a reference. This greater adherence was 33% higher in areas outside the capitals when compared to the prevalence among adolescents who lived in the capitals. Adolescents who declared themselves to be very happy with their bodies had an outcome prevalence 172% higher compared to those who were very unhappy, and the prevalence of this greater adherence was also higher among those who considered their own health to be poor (511%) or very good (287%), with the category “very bad” serving as a reference.

## Discussion

The prevalence of greater adherence to Guidelines recommendations by adolescents was zero (more restricted model) or very low (less restricted model). The difficulty in adhering to Guidelines recommendations by adolescents caught attention, as the markers for eating practices evaluated in PeNSE 2019 are more qualitative in nature and did not consider information on the quantity of food consumed by adolescents. The presence of quantitative measures of food consumption would perhaps further reduce the prevalence of those adolescents who satisfactorily meet dietary and nutritional guidelines. Furthermore, greater adherence was mainly associated with territorial markers of less urbanization - living in rural areas, in the Northeast and the Midwest regions, in areas outside the capitals - and with behaviors of better perception of body image and very good or poor health. It is important to note that this last variable and the “bad health” category need to be investigated carefully in other studies, as the estimates demonstrate little precision due to the wide range of confidence intervals and the paradoxical situation of the outcome proportions being higher in groups of adolescents with these positive and negative health markers.

The difficulty in adhering to Guidelines recommendations was highlighted in a previous study with adolescents from the South region, where less than 0.2% of participants managed to meet all six recommendations proposed by the 2005 version. The goal of achieving the ten recommendations of the previous Brazilian guide, which were based on the number of servings, was not achieved by any of the participants (16).

The multivariate analysis of this study showed that adherence to Guidelines recommendations is positively associated with less urbanized areas, where family relationships and traditional eating practices are commonly more present. These results suggest that lower urbanization favors the adoption of healthier eating habits. Therefore, it is of utmost importance to develop public policies capable of nurturing traditional cultures, promoting healthy food environments based on the recognition of the role of the territories where one lives in promoting health-promoting eating practices (17).

It is known that the implementation of effective public policies to promote healthy eating habits requires consideration of the particularities of each setting, given the complexity of food systems (18). The environment where eating occurs, especially in urban areas, exerts a great influence on individuals’ food choices. Factors such as advertising, information and food prices play a crucial role in this process, and can either facilitate or hinder the adoption of healthier habits (19). Therefore, creating strategies that promote healthier food environments, considering the specificities of each location, is greatly needed.

Urban and peri-urban agriculture emerges, for example, as a strategy to promote food security in Brazil. By involving adolescents and young people, programs that promote this type of agricultural production can foster healthier eating habits and the transition to more sustainable food systems (20).

A positive perception of body and health proved to be a relevant factor for greater adherence to Guidelines recommendations among adolescents, regardless of sociodemographic characteristics. This association highlights the importance of addressing dietary and nutritional issues during adolescence in an holistic manner, considering the physical, psychological and social changes typical of this stage of life (21). Dissatisfaction with body weight and distorted body image are common problems in adolescence and are associated with health risk behaviors, such as risky eating practices for eating disorders and low self-esteem (22,23). Therefore, interventions that promote body acceptance and self-esteem can be effective in promoting healthy eating habits and preventing diet-related health problems.

In this context, a longitudinal study with Norwegian adolescents demonstrated that concern about body and weight is associated with worse self-rated health and self-esteem, while a positive self-perception predicts a more favorable nutritional status (24). This evidence suggests that promoting body acceptance and self-esteem may be an effective strategy for improving eating practices, as well as preventing obesity and eating disorders.

Positive self-rated health is required for the promotion of healthy eating among adolescents, as it is associated, for example, with greater consumption of fruits and vegetables (25). Furthermore, previous studies have shown that individuals with better self-perceived health tend to exhibit healthier lifestyle behaviors (26). However, the results of this study suggest a paradoxical and more complex relationship, since in addition to adolescents with better self-rated health showing greater adherence to the multiple Guidelines recommendations, those who self-rated their health as poor also associated themselves with this set of positive eating practices.

This apparent contradiction can be explained by the fact that the perception of vulnerability to disease motivates, in some cases, the adoption of healthier practices, as a way of treating a current condition or preventing future health problems (27). As highlighted previously, this direct association should be considered with caution, as the PR estimates in the present study also show little precision, despite the statistical difference identified. However, this apparently controversial result should be considered for the development of new longitudinal quantitative and qualitative studies to better understand this phenomenon. Practical initiatives of food and nutritional care for adolescents implemented in health and education services show that experiencing illness by this public sometimes leads to a change in eating practices, with the incorporation of food and nutritional guidelines. 

The school environment is strategic for promoting healthy eating among adolescents, but actions in this regard need to be qualified in public and private schools. It is noteworthy that no differences were found according to the type of school administration (public or private). Given that the PNAE was restructured in 2020 to align with the Guidelines and that the data in the article are from 2019, it is expected that such changes may be identified in the results of future PeNSE studies. Furthermore, achieving more effective changes in the eating habits of this age group depends on incorporating national guidelines capable of guiding the provision of healthy foods in the context of the PNAE, such as those that consider local culture and Guidelines recommendations, also in school cafeterias. To this end, the participation of the school community (managers, teachers, nutritionists, parents and students) and of different sectors (education, health, commerce) is essential for the success of these initiatives (28).

Aligned with the Food and Nutrition Education Reference Framework (29) and Law No. 13.666/2018 (30), actions that promote healthy eating in schools can significantly contribute to improving adherence to Guidelines among adolescents. Decree No. 11,821/2023 strengthens this perspective by highlighting food and nutritional education, food donation and marketing, and marketing communication as strategic axes for promoting healthy food environments in schools (31).

A point to be argued about in terms of the results of this study is the use of markers of dietary practices that were not developed to accurately measure adherence to the Guidelines, especially considering that six steps recommended by this guide could not be assessed based on the questions in PeNSE 2019. However, the representativeness of the data for the population of Brazilian adolescent students and the robustness of the IBGE research highlight the importance of the evidence this research makes clear. It is worth noting that, as this is a cross-sectional study, there are some limitations inherent to this epidemiological design, such as: not establishing cause and effect relationships, not monitoring the population over time, and being susceptible to confounding variables that were not collected. Such methodological weaknesses may have generated the paradoxical result of self-perceived health discussed previously. Therefore, the findings of this research must be carefully verified, due to the limitations inherent to this type of study.

Finally, it was found that greater adherence to multiple Guidelines recommendations among adolescent students in Brazil was associated with factors marked by less urbanized areas in the country and with perceptions that were mainly positive about their body and health. It is important to note that the paradoxical association of the outcome with very good and poor self-rated health should not be disregarded and needs to be explored in future studies with this audience. Therefore, interventions to improve the eating practices of this age group must be based on the importance of these territorial and behavioral contexts for the development of more effective actions.

## Data Availability

The database and the codes used for the analysis in the research are available at https://www.ibge.gov.br/estatisticas/downloads-estatisticas.html?caminho=pense/2009/microdados/.
