# Supplementary material for: Adherence to multiple recommendations of the Dietary Guidelines for the Brazilian Population (Guia Alimentar para a População Brasileira) and associated factors among adolescents: a cross-sectional study with data from the National Students’ Health Survey, 2019
Source: Epidemiol Serv Saude. 2025 Jun 13;34:e20240404. doi: 10.1590/S2237-96222025v34e20240404.en (PMC12176437; doi:10.1590/S2237-96222025v34e20240404.en)
Supplement: Translation

# Translation: Adesão a múltiplas recomendações do *Guia Alimentar para População Brasileira* e fatores associados entre adolescentes: estudo transversal com dados da Pesquisa Nacional de Saúde do Escolar, 2019

**DOI:** 10.1590/S2237-96222025v34e20240404.pt

**Authors:** Diôgo Vale, Maria Eduarda da Costa Andrade, Natalie Marinho Dantas

**Affiliations:** 1 Instituto Federal de Educação, Ciência e Tecnologia do Rio Grande do Norte, Ceará-Mirim, RN, Brazil; 2 Instituto Federal de Educação, Ciência e Tecnologia do Rio Grande do Norte, Natal, RN, Brazil; 3 Universidade Estadual de Campinas, Faculdade de Engenharia de Alimentos, Departamento de Ciência de Alimentos e Nutrição, Campinas, SP, Brazil

**Keywords:** Adolescente, Guias Alimentares, Dieta, Fatores Sociais, Estudos Transversais

## Abstract

**Objetivo:**

Analisar as prevalências de adesão a múltiplas recomendações do *Guia Alimentar para População Brasileira* entre adolescentes escolares do Brasil em 2019 e associações com fatores sociodemográficos e comportamentais.

**Métodos:**

Estudo transversal com microdados da Pesquisa Nacional de Saúde do Escolar de 2019. A variável dependente foi a adesão a múltiplas recomendações do Guia, com base nos marcadores de práticas alimentares. As variáveis ​​independentes foram sexo, raça/cor da pele, escolaridade materna, área de residência, região geográfica, administração da escola, território de moradia, utilização de medidas laxativas, de fórmulas para perda ou ganho de peso, satisfação corporal e autopercepção de saúde. Razões de prevalências (RP) e intervalos de confiança de 95% (IC95%) foram calculados por regressão de Poisson.

**Resultados:**

A prevalência de melhor adesão a múltiplas recomendações do Guia entre adolescentes brasileiros foi de 0,4% (IC95% 0,3; 0,4). Essa adesão às recomendações teve suas proporções elevadas pelos fatores: morar em áreas rurais (RP 1,91; IC95% 1,16; 3,13), na região Nordeste (RP 1,92; IC95% 1,10; 3,35) e Centro-Oeste (RP 2,29; IC95% 1,28; 4,09), em territórios fora das capitais (RP 1,33; IC95% 1,01; 1,77), declarar-se muito satisfeito com o corpo (RP 2,72; IC95% 1,20; 6,20) e considerar a própria saúde como muito boa (RP 3,87; IC95% 1,12; 13,43) ou ruim (RP 6,11; IC95% 1,20; 31,12).

**Conclusão:**

A prevalência de adesão a múltiplas recomendações do Guia entre adolescentes é muito baixa e os fatores associados com a melhor adesão caracterizam-se por territórios menos urbanizados e, principalmente, com as percepções positivas sobre o corpo e a saúde.

**Aspectos éticos**

A presente pesquisa respeitou os princípios éticos e para as análises foi utilizado o banco de microdados da Pesquisa Nacional de Saúde do Escolar de (PeNSE 2019) disponibilizado pelo Instituto Brasileiro de Geografia e Estatística. A PeNSE 2019 foi aprovada por meio do Parecer Conep n^o^ 3.249.268, de 8.4.2019.:

## Introdução

A alimentação inadequada e o excesso de peso na adolescência representam um desafio para a saúde pública brasileira, elevando significativamente as incidências e prevalências de doenças crônicas não transmissíveis. Estudos recentes demonstram um aumento preocupante nas prevalências de sobrepeso e obesidade nessa população, tornando urgente a implementação de medidas eficazes para reverter esse cenário (1,2).

O consumo excessivo de alimentos ultraprocessados (27% da energia em 2017-2018) e o baixo consumo de alimento in natura ou minimamente processados, como de frutas, verduras e legumes na alimentação de adolescentes brasileiros (3,4) são problemas complexos, influenciados por fatores sociais e emocionais (5,6).

Diante dessas problemáticas, o Ministério da Saúde brasileiro tem atuado para promover a alimentação saudável e combater o aumento do sobrepeso e da obesidade. Nesse sentido, foram publicados o Plano de Ações Estratégicas para o Enfrentamento das Doenças Crônicas e Agravos não Transmissíveis no Brasil 2021-2030 (7) e o *Guia Alimentar para a População Brasileira* (8). Este último, com diretrizes claras, serviu de base para iniciativas como o Protocolo de uso do Guia na adolescência para o aconselhamento nutricional na Atenção Básica (9) e a Resolução 6/2020 do Programa Nacional de Alimentação Escolar (PNAE) que buscou fortalecer a alimentação saudável para esse público no contexto das escolas (10).

Apesar das iniciativas governamentais, como a publicação do Guia (8), que enfatiza práticas alimentares saudáveis e sustentáveis, ainda há uma lacuna significativa no conhecimento sobre a adesão de adolescentes brasileiros às suas recomendações. O Guia aborda não apenas a escolha dos alimentos, mas os aspectos socioculturais da alimentação, como comer com atenção e em companhia. No entanto, a influência desses fatores socioculturais na prática alimentar dos adolescentes e como eles aplicam as recomendações do Guia ainda são pouco explorados (11).

Sabe-se que o consumo de alimentos ultraprocessados por adolescentes varia de acordo com fatores sociodemográficos (12,13). No entanto, são necessárias pesquisas considerando aspectos sociais e comportamentais, como insatisfação corporal e comportamentos de risco para transtornos alimentares, que se associam a maior ou menor adesão às recomendações do Guia.

Com isso, este estudo visa preencher essa lacuna, analisando as prevalências de adesão a múltiplas recomendações do Guia entre adolescentes escolares do Brasil em 2019 e associações com fatores sociodemográficos e comportamentais. Os resultados desta pesquisa contribuirão para o dimensionamento das potencialidades e dos desafios na utilização desse guia para promoção da alimentação adequada e saudável com esse grupo etário.

## Métodos

### Delineamento do estudo e fonte de dados

Este estudo caracterizou-se como transversal e observacional do tipo inquérito de base escolar (dados individuados). A fonte de dados desta pesquisa quantitativa, descritiva e analítica foi a Pesquisa Nacional de Saúde do Escolar de 2019 (PeNSE 2019). Essa foi a quarta edição dessa pesquisa de base escolar desenvolvida pelo Instituto Brasileiro de Geografia e Estatística. A PeNSE consiste em uma pesquisa de base escolar que coleta informações sobre a saúde de adolescentes brasileiros, sendo fundamental para vigilância alimentar e nutricional desse grupo etário (14).

### Contexto, participantes e tamanho do estudo

A amostra da PeNSE 2019 foi por conglomerados, probabilística e composta por estudantes de 13 a 17 anos, matriculados e com frequência regular em escolas públicas e privadas de todo o território nacional. A seleção das escolas seguiu o delineamento em dois estágios, com estratificação por região e porte da escola. Coletaram-se dados de 4.242 escolas e 6.612 turmas, resultando em amostra final de 159.245 adolescentes com questionários válidos (14).

A coleta de dados foi realizada entre 9 de abril e 30 de setembro de 2019 por meio de questionário autoaplicável com os estudantes adolescentes em sala de aula. O questionário abordou temas relacionados à saúde dos adolescentes, incluindo hábitos alimentares, atividade física, saúde mental, saúde sexual e reprodutiva, entre outros (14).

A PeNSE foi aprovada pelo Comitê Nacional de Ética em Pesquisa com o registro nº 3.249.268 de 8 de abril de 2019 (14). O presente trabalho atende aos preceitos éticos dispostos na resolução nº 510, de 7 de abril de 2016, do Conselho Nacional de Saúde (15).

O presente estudo apresenta resultados relativos a 153.153 adolescentes, o que corresponde a 96,2% da amostra inicial da PENSE 2019. Para garantir a consistência das análises, 3,8% dos indivíduos da amostra inicial foram categorizados como “ausente” (99), quando apresentavam dado omisso para alguma das variáveis de práticas alimentares que originaram a “variável dependente”. Essa abordagem foi adotada para manter as ponderações do banco de dados original, assegurando a preservação do efeito adequado do desenho amostral.

### Variáveis

A variável dependente foi a adesão a múltiplas recomendações do Guia. Essa variável foi construída pelos autores do estudo a partir dos 22 marcadores de práticas alimentares disponíveis no banco de microdados da PeNSE 2019. Inicialmente, esses marcadores foram distribuídos segundo os “dez passos para uma alimentação adequada e saudável” presentes no Guia (8). Nesse processo, foram identificados marcadores que caracterizam diretamente apenas quatro passos do Guia: (a) fazer de alimentos in natura ou minimamente processados a base da alimentação; (b) evitar o consumo de alimentos ultraprocessados; (c) comer com regularidade e atenção, em ambientes apropriados e, sempre que possível, com companhia; e (d) dar preferência, quando fora de casa, a locais que servem refeições feitas na hora (quadro 1).

Posteriormente, cada marcador de prática alimentar teve suas respostas categorizadas em “0” (não atendia a recomendação do Guia) e “1” (atendia a recomendação do Guia). A avaliação da adesão a múltiplas recomendações do Guia envolveu a utilização da pontuação total resultante da somatória dos marcadores: 1.1, 1.2, 1.3, 2.1, 2.2, 2.3, 3.1, 3.2, 3.3 e 4.1. Com isso, essa variável dependente assumiu valores de “0” até “10” (Tabela 1).

**Tabela 1:** Marcadores de práticas alimentares da Pesquisa Nacional de Saúde do Escolar 2019 segundo recomendações do *Guia Alimentar para População Brasileira* para construção das variáveis de adesão a múltiplas recomendações do Guia entre adolescentes. Brasil, 2019

Recomendações do *Guia Alimentar para População Brasileira* com marcadores diretos disponíveis na Pesquisa Nacional de Saúde do Escolar	Múltipla adesão a recomendações do Guia com categorias de práticas alimentares recomendadas ideais (mais restrito)	Múltipla adesão a recomendações do Guia com categorias de práticas alimentares recomendadas possíveis (menos restrito)
**1 Fazer de alimentos in natura ou minimamente processados a base da alimentação**		
1.1 Nos últimos 7 dias, em quantos dias você comeu feijão?	0-6 dias/semana [0] 7 dias/semana [1]	0-6 dias/semana [0] 7 dias/semana [1]
1.2 Nos últimos 7 dias, em quantos dias você comeu frutas frescas ou salada de frutas?	0-6 dias/semana [0] 7 dias/semana [1]	0-6 dias/semana [0] 7 dias/semana [1]
1.3 Nos últimos 7 dias, em quantos dias você comeu pelo menos um tipo de legume ou verdura que não seja batata ou aipim (mandioca/macaxeira)?	0-6 dias/semana [0] 7 dias/semana [1]	0-6 dias/semana [0] 7 dias/semana [1]
**2 Evitar o consumo de alimentos ultraprocessados**		
2.1 Nos últimos 7 dias, em quantos dias você tomou refrigerante?	1-7 dias/semana [0] 0 dias/semana [1]	3-7 dias/semana (0) 2 dias ou menos/semana (1)
2.2 Nos últimos 7 dias, em quantos dias você comeu guloseimas doces, como balas, confeitos, chocolates, chicletes, bombons, pirulitos e outros?	1-7 dias/semana [0] 0 dias/semana [1]	3-7 dias/semana (0) 2 dias ou menos/semana (1)
2.3 Somatória do consumo de alimentos ultraprocessados no dia anterior à pesquisa (2.3.1 até 2.3.13)	Consumiu 1-13 alimentos [0] Não consumiu nenhum alimento [1]	Consumiu 4-13 alimentos [0] Consumiu 3 itens ou menos no dia anterior - percentil 25 [1]
2.3.1 Ontem, você tomou refrigerante?	Não [0] Sim [1]	Não [0] Sim [1]
2.3.2 Ontem, você tomou suco de fruta em caixinha ou lata?	Não [0] Sim [1]	Não [0] Sim [1]
2.3.3 Ontem, você tomou refresco em pó?	Não [0] Sim [1]	Não [0] Sim [1]
2.3.4 Ontem, você tomou bebida achocolatada?	Não [0] Sim [1]	Não [0] Sim [1]
2.3.5 Ontem, você tomou iogurte com sabor?	Não [0] Sim [1]	Não [0] Sim [1]
2.3.6 Ontem, você comeu salgadinho de pacote (chips) ou biscoito/bolacha salgado?	Não [0] Sim [1]	Não [0] Sim [1]
2.3.7 Ontem, você comeu biscoito ou bolacha doce, biscoito recheado ou bolinho de pacote?	Não [0] Sim [1]	Não [0] Sim [1]
2.3.8 Ontem, você comeu chocolate, sorvete, gelatina, flan ou outra sobremesa industrializada?	Não [0] Sim [1]	Não [0] Sim [1]
2.3.9 Ontem, você comeu salsicha, linguiça, mortadela ou presunto?	Não [0] Sim [1]	Não [0] Sim [1]
2.3.10 Ontem, você comeu pão de forma, pão de cachorro-quente ou pão de hambúrguer?	Não [0] Sim [1]	Não [0] Sim [1]
2.3.11 Ontem, você comeu margarina?	Não [0] Sim [1]	Não [0] Sim [1]
2.3.12 Ontem, você comeu maionese, ketchup ou outros molhos industrializados?	Não [0] Sim [1]	Não [0] Sim [1]
2.3.13 Ontem, você comeu macarrão instantâneo (miojo), sopa de pacote, lasanha congelada ou outro prato pronto comprado congelado?	Não [0] Sim [1]	Não [0] Sim [1]
**3 Comer com regularidade e atenção, em ambientes apropriados e, sempre que possível, com companhia**		
3.1 Você costuma tomar o café da manhã?	0-6 dias/semana [0] 7 dias/semana [1]	0-6 dias/semana (0) 7 dias/semana (1)
3.2 Você costuma almoçar ou jantar com sua mãe, pai ou responsável?	0-6 dias/semana [0] 7 dias/semana [1]	0-6 dias/semana (0) 7 dias/semana (1)
3.3 Nas suas refeições, com que frequência você costuma comer fazendo alguma outra coisa (assistindo à TV, mexendo no computador ou no celular)?	1 ou mais dias/semana [0] Nunca [1]	3 ou mais dias/semana [0] 2 dias ou menos [1]
**4 Dar preferência, quando fora de casa, a locais que servem refeições feitas na hora**		
4.1 Nos últimos 7 dias, em quantos deles você comeu em lanchonetes, barracas de cachorro quente, pizzaria, fast food etc.?	1 ou mais dias/semana [0] 0 dias/semana [1]	3 ou mais dias/semana [0] 2 dias ou menos [1]
Recomendações do *Guia Alimentar para População Brasileira* sem marcadores diretos disponíveis na Pesquisa Nacional de Saúde do Escolar		
Utilizar óleos, gorduras, sal e açúcar em pequenas quantidades ao temperar e cozinhar alimentos e criar preparações culinárias		
Limitar o consumo de alimentos processados		
Fazer compras em locais que ofertem variedades de alimentos in natura ou minimamente processados		
Desenvolver, exercitar e partilhar habilidades culinárias		
Planejar o uso do tempo para dar à alimentação o espaço que ela merece		
Ser crítico quanto a informações, orientações e mensagens sobre alimentação veiculadas em propagandas comerciais		

^a^Números entre chaves representam valores pontuais atribuídos a cada categoria para o cálculo dos dois modelos de múltipla adesão.

Considerando as posteriores etapas de análises estatísticas, essa variável principal foi calculada de duas maneiras: (a) múltipla adesão a recomendações do Guia, com categorias de práticas alimentares “recomendadas ideais” (mais restrita), caracterizada, por exemplo, pelo consumo de alimentos in natura e/ou minimamente processados em todos os dias da semana e ausência do consumo de alimentos ultraprocessados; e (b) múltipla adesão a recomendações do Guia com categorias de práticas alimentares “recomendadas possíveis” (menos restrita), cujo consumo de alimentos ultraprocessados, por exemplo, foi tolerado até duas vezes na semana. A criação das duas maneiras de calcular essa variável ocorreu diante da impossibilidade de realizar modelagens com a categoria mais restrita (a) (Tabela 2).

**Tabela 2:** Prevalências (%) e intervalos de confiança de 95% (IC95%) da múltipla adesão a múltiplas recomendações do *Guia Alimentar para População Brasileira* de acordo com categorias recomendadas ideais (mais restrito) e possíveis (menos restrito) entre adolescentes escolares. Brasil, 2019 (n=153.153^a^)

Número de práticas alimentares de adesão ao Guia	Adesão a recomendações do Guia com categorias de práticas alimentares recomendadas ideais (mais restrito)		Adesão a recomendações do Guia com categorias de práticas alimentares recomendadas possíveis (menos restrito)	
	%	IC95%	%	IC95%
0	4,6	4,4; 4,8	1,1	0,9; 1,2
1	11,8	11,5; 12,2	4,1	3,9; 4,4
2	18,8	18,4; 19,3	9,3	9,0; 9,6
3	21,4	21,0; 21,9	15,4	15,1; 15,8
4	18,9	18,5; 19,3	19,5	19,1; 19,9
5	13,3	12,9; 13,6	19,3	18,8; 19,7
6	6,8	6,5; 7,0	15,5	15,1; 15,9
7	3,0	2,8; 3,2	9,6	9,3; 9,9
8	1,1	1,0; 1,2	4,4	4,2; 4,6
9	0,2	0,2; 0,3	1,5	1,4; 1,6
10 (maior adesão)	0,0	0,0; 0,1	0,4	0,3; 0,4

^a^Número de adolescentes com dados coletados e válidos para este estudo. Ao considerar o efeito da amostra, os dados representam 15.192.972 adolescentes brasileiros.

A criação desses modelos a priori deu-se com base nas recomendações qualitativas do Guia que não apresenta referências sobre quantidades e frequência de práticas alimentares, mas recomenda a incorporação dessas práticas promotoras de nutrição e saúde concomitantemente a de um padrão de vida mais saudável (8).

As variáveis ​​independentes consideradas neste estudo foram de duas dimensões:

- fatores sociodemográficos - sexo (masculino e feminino), raça/cor da pele autodeclarada (branca, preta, parda, amarela e indígena), escolaridade materna (Não estudou, Ensino fundamental incompleto, Ensino fundamental completo, Ensino médio incompleto, Ensino médio completo, Ensino superior incompleto, Ensino superior completo), área de residência (urbana e rural), região geográfica (Norte, Nordeste, Sudeste, Sul e Centro-Oeste), administração da escola (privada e pública), e o território de moradia (capital, não capital);
- fatores comportamentais - utilização de medidas laxativas (sim, não), utilização de fórmulas para perda de peso sem acompanhamento profissional (sim, não), utilização de fórmulas para ganho de peso ou massa muscular sem acompanhamento profissional (sim, não), satisfação corporal (muito insatisfeito, insatisfeito, indiferente, satisfeito, muito satisfeito), e autopercepção de saúde (muito boa, boa, regular, ruim, muito ruim).

### Viés

Para minimizar possíveis vieses, o estudo adotou diversas medidas metodológicas. Foram utilizados os estimadores de amostragem da PeNSE 2019 para ajustar os resultados e garantir a representatividade da amostra. A variável de adesão ao Guia proposta pelos autores foi construída de forma sistemática e rigorosa, com base nas recomendações do guia e nas variáveis disponíveis na pesquisa. Além disso, a categorização das variáveis foi revisada por três pesquisadores da área para garantir a consistência e a confiabilidade dos dados.

### Métodos estatísticos

As prevalências e os respectivos intervalos de confiança de 95% (IC95%) foram estimados para a (a) múltipla adesão a recomendações do Guia com categorias de práticas alimentares recomendadas ideais (mais restrito) e para a (b) múltipla adesão a recomendações do Guia com categorias de práticas alimentares recomendadas possíveis (menos restrito).

Para as regressões bivariadas e multivariadas de Poisson, foi utilizada a variável categórica dicotômica de adesão a múltiplas recomendações do Guia. Essa foi construída considerando a (b) múltipla adesão a recomendações do Guia com categorias de práticas alimentares recomendadas possíveis (menos restrito). Com esses resultados, aqueles adolescentes que pontuaram de zero a nove ficaram na categoria de referência (alguma adesão ao Guia com valor “zero”) e aqueles que pontuaram para as dez práticas foram categorizados como maior adesão ao Guia com valor “um”. A decisão por categorizar “0” como “alguma adesão” se deu, pois, mesmo o(a) adolescente não tendo atingido o modelo possível a priori, tinham alguma prática alimentar positiva incorporada na sua rotina segundo os dados coletados. Sabe-se que tais ações positivas também precisam ser valorizadas nos grupos populacionais, pois o Guia não determina frequências semanais em sua publicação (8). 

As regressões bivariadas e multivariadas de Poisson foram aplicadas para avaliar as associações da variável dependente com as variáveis independentes (fatores sociodemográficos e comportamentais) a partir de estimativas das Razões de Prevalência (RP) e respectivos IC95%. O método Backward foi empregado para a construção do modelo de regressão multivariada, onde as variáveis ​​relacionadas a um nível de significância estatística menor que 20% na análise bivariada foram todas incluídas e, posteriormente, aquelas que não atingiram o nível determinando de significância foram removidas uma a uma. Apenas permaneceram no modelo final (multivariado) aquelas variáveis que apresentaram p-valor<0,05. Essas análises foram conduzidas usando o programa Stata, versão 14.2, com o módulo Survey considerando os estimadores do desenho amostral da PeNSE 2019.

## Resultados

Dentre os adolescentes avaliados, a maioria era do sexo feminino (51,4%), se declarava da raça/cor da pele branca (36,0%) e morava na região Sudeste (40,2%) do Brasil.

Entre adolescentes escolares brasileiros, ninguém (0,0%; IC95% 0,0; 0,1) conseguiu atingir as dez práticas alimentares avaliadas para caracterizar a múltipla adesão a recomendações do Guia com categorias ideais (mais restrito). Ao estimar a múltipla adesão a recomendações do Guia com categorias de práticas alimentares recomendadas possíveis (menos restrito), 0,4% (IC95% 0,3; 0,4) dos adolescentes apresentaram maior adesão múltipla a recomendações do Guia (Tabela 2).

Quanto aos fatores sociodemográficos e comportamentais associados e que elevaram as prevalências da maior adesão a múltiplas recomendações do Guia entre adolescentes, compuseram o modelo de Poisson multivariado: morar em áreas rurais (RP 1,91; IC95% 1,16; 3,13), na região Nordeste (RP 1,92; IC95% 1,10; 3,35) e Centro-Oeste (RP 2,29; IC95% 1,28; 4,09), em territórios fora das capitais (RP 1,33; IC95% 1,01; 1,77), declarar-se muito satisfeito com o corpo (RP 2,72; IC95% 1,20; 6,20) e considerar a própria saúde como ruim (RP 6,11; IC95% 1,20; 31,12) ou muito boa (RP 3,87; IC95% 1,12; 13,43) (Tabela 3).

**Tabela 3:** Prevalências (%), Razões de Prevalência (RP) e intervalos de confiança de 95% (IC95%) da maior ou alguma adesão a múltiplas recomendações a categorias de práticas alimentares recomendadas possíveis (menos restrito) do *Guia Alimentar para População Brasileira* entre adolescentes escolares segundo fatores sociodemográficos e comportamentais. Brasil, 2019 (n=153.153^a^)

Variável	Maior adesão a múltiplas recomendações do Guia % (IC95%)	Alguma adesão a múltiplas recomendações do Guia % (IC95%)	p-valor	Modelo bivariado		Modelo multivariado
				RP (IC95%)	p-valor	RP (IC95%) p-valor
Sexo			0,906			
Masculino	0,4 (0,3; 0,4)	99,6 (99,6; 99,7)				
Feminino	0,4 (0,3; 0,5)	99,6 (99,5; 99,7)				
**Área de residência**			0,001		<0,001	
Urbana	0,3 (0,3; 0,4)	99,7 (99,6; 99,7)		Ref		Ref
Rural	0,8 (0,5; 1,2)	99,2 (98,8; 99,5)		2,50 (1,55; 4,04)		1,91 (1,16; 3,13) 0,011
**Raça/cor da pele**			0,742			
Branca	0,4 (0,3; 0,5)	99,6 (99,5; 99,7)				
Preta	0,3 (0,2; 0,5)	99,7 (99,5; 99,8)				
Amarela	0,4 (0,2; 0,8)	99,6 (99,2; 99,8)				
Parda	0,4 (0,3; 0,5)	99,6 (99,5; 99,7)				
Indígena	0,2 (0,1; 0,7)	99,8 (99,3; 99,9)				
**Escolaridade materna**			0,254			
Não estudou	0,6 (0,2; 1,3)	99,4 (98,7; 99,8)				
Ensino fundamental incompleto	0,3 (0,2; 0,5)	99,7 (99,5; 99,8)				
Ensino fundamental completo	0,5 (0,3; 1,0)	99,5 (99,0; 99,7)				
Ensino médio incompleto	0,3 (0,1; 0,5)	99,7 (99,5; 99,9)				
Ensino médio completo	0,2 (0,1; 0,4)	99,8 (99,6; 99,9)				
Ensino superior incompleto	0,3 (0,1; 0,6)	99,7 (99,4; 99,9)				
Ensino superior completo	0,4 (0,3; 0,6)	99,6 (99,4; 99,7)				
**Região Geográfica**			0,026			
Sul	0,2 (0,1; 0,3)	99,8 (99,7; 99,9)		Ref		Ref
Sudeste	0,3 (0,2; 0,5)	99,7 (99,5; 99,8)		1,50 (0,74; 3,02)	0,260	1,53 (0,78; 3,02) 0,216
Norte	0,3 (0,2; 0,5)	99,7 (99,5; 99,8)		1,46 (0,77; 2,77)	0,244	1,16 (0,59; 2,89) 0,048
Centro-Oeste	0,5 (0,3; 0,6)	99,5 (99,4; 99,7)		2,27 (1,24; 4,14)	0,008	2,29 (1,28; 4,09) 0,005
Nordeste	0,5 (0,4; 0,6)	99,5 (99,4; 99,6)		2,25 (1,24; 4,06)	0,007	1,92 (1,10; 3,35) 0,021
**Administração da escola**			0,066			
Privada	0,3 (0,2; 0,3)	99,7 (99,7; 99,8)		Ref		
Pública	0,4 (0,3; 0,4)	99,6 (99,6; 99,7)		1,30 (0,92; 1,82)	0,137	
**Território de moradia**			0,008			
Capital	0,2 (0,2; 0,3)	99,8 (99,7; 99,8)		Ref		Ref
Não capital	0,4 (0,3; 0,5)	99,6 (99,5; 99,7)		1,54 (1,15; 1,06)	0,004	1,33 (1,01; 1,77) 0,048
**Medidas laxativas**			0,045			
Não	0,4 (0,3; 0,4)	99,6 (99,6; 99,7)		Ref		
Sim	0,2 (0,1; 0,4)	99,8 (99,6; 99,9)		0,48 (0,23; 0,99)	0,048	
**Fórmula para perda de peso**			0,556			
Não	0,3 (0,3; 0,4)	99,7 (99,6; 99,7)				
Sim	0,5 (0,2; 1,2)	99,5 (98,8; 99,8)				
**Fórmula para ganho de peso**			0,126			
Não	0,4 (0,3; 0,4)	99,6 (99,6; 99,7)		Ref		
Sim	0,2 (0,1; 0,4)	99,8 (99,6; 99,9)		0,49 (0,19; 1,26)	0,140	
**Satisfação corporal**			<0,001			
Muito insatisfeito	0,2 (0,1; 0,3)	99,8 (99,7; 99,9)		Ref		Ref
Insatisfeito	0,1 (0,1; 0,2)	99,9 (99,8; 99,9)		0,56 (0,25; 1,25)	0,154	0,61 (0,27; 1,37) 0,232
Indiferente	0,1 (0,1; 0,3)	99,9 (99,7; 99,9)		0,82 (0,42; 1,59)	0,558	0,92 (0,46; 1,85) 0,820
Satisfeito	0,3 (0,2; 0,4)	99,7 (99,6; 99,8)		1,72 (0,79; 3,77)	0,174	1,76 (0,79; 3,93) 0,168
Muito satisfeito	0,7 (0,5; 0,8)	99,3 (99,2; 99,5)		3,71 (1,80; 7,68)	<0,001	2,72 (1,20; 6,20) 0,017
**Autopercepção do estado geral de saúde**			<0,001			
Muito ruim	0,2 (0,1; 0,4)	99.8 (99,6; 99,9)		Ref		Ref
Ruim	0,6 (0,2; 1,6)	99,4 (98,4; 99,8)		3,61 (0,83; 15,68)	0,087	6,11 (1,20; 31,12) 0,029
Regular	0,2 (0,1; 0,2)	99,8 (99,8; 99,9)		0,99 (0,31; 3,15)	0,991	1,45 (0,38; 5,46) 0,585
Bom	0,2 (0,1; 0,3)	99,8 (99,7; 99,9)		1,35 (0,44; 4,10)	0,595	1,68 (0,47; 6,05) 0,427
Muito bom	0,6 (0,5; 0,8)	99,4 (99,2; 99,5)		4,15 (1,41; 12,26)	0,010	3,87 (1,12; 13,43) 0,033

^a^Número de adolescentes com dados coletados e válidos para este estudo. Ao considerar o efeito da amostra, os dados representam 15.192.972 adolescentes brasileiros.

Na prática, os valores dessas RP no modelo multivariado representam elevações nas prevalências do desfecho em relação aos fatores sociodemográficos e comportamentais que permaneceram associados. A prevalência de maior adesão Guia entre adolescentes foi 91% mais elevada entre aqueles que moravam em áreas rurais comparada à prevalência desse desfecho em áreas urbanas. A prevalência desse tipo de adesão ao Guia também foi 92% maior na região Nordeste, e 129%, no Centro-Oeste tendo a prevalência do Norte como referência. Essa maior adesão foi 33% mais elevada nos territórios fora das capitais quando comparado à prevalência entre adolescentes que moravam nas capitais. Adolescentes que se declararam muito satisfeitos com o corpo tiveram prevalência do desfecho 172% maior comparado aos muito insatisfeitos, e a prevalência dessa maior adesão também foi mais elevada entre aqueles que consideravam a própria saúde como ruim (511%) ou muito boa (287%) tendo a categoria “muito ruim” como referência.

## Discussão

As prevalências de maior adesão a recomendações do Guia por adolescentes foram nulas (modelo mais restrito) ou muito baixas (modelo menos restrito). A dificuldade de adesão a recomendações do Guia por adolescentes chamou atenção, pois os marcadores de práticas alimentares avaliados na PeNSE 2019 têm caráter mais qualitativo e não consideravam informações sobre quantidade de alimentos consumidos pelos adolescentes. A presença de medidas quantitativas do consumo alimentar talvez diminuísse ainda mais as prevalências daqueles adolescentes que atingem de maneira satisfatória as orientações alimentares e nutricionais. Além disso, a maior adesão associou-se, principalmente, a marcadores territoriais de menor urbanização - morar em áreas rurais, na região Nordeste e Centro-Oeste, em territórios fora das capitais - e a comportamentos de melhor percepção de imagem corporal e de saúde muito boa ou ruim. Ressalta-se que essa última variável e a categoria “ruim” precisa ser investigada com atenção em outros estudos, pois as estimativas demonstram pouca precisão devido a grande amplitude dos intervalos de confiança e a situação paradoxal das proporções do desfecho serem mais elevados nos grupos de adolescentes com esses marcadores positivos e negativos de saúde.

A dificuldade de aderir às recomendações do Guia foi evidenciada em um estudo anterior com adolescentes da região Sul, onde menos de 0,2% dos participantes conseguiram atender a todas as seis recomendações propostas pela versão de 2005. A meta de atingir as dez recomendações do guia brasileiro anterior, que eram baseadas no número de porções, não foi alcançada por nenhum dos participantes (16).

A análise multivariada deste estudo apontou que a adesão às recomendações do Guia está positivamente associada a características dos territórios menos urbanizados, onde, comumente, as relações familiares e as práticas alimentares tradicionais são mais presentes. Esses resultados sugerem que a menor urbanização favorece a adoção de hábitos alimentares mais saudáveis. Portanto, é fundamental desenvolver políticas públicas que valorizem as culturas tradicionais, promovam ambientes alimentares saudáveis a partir do reconhecimento do papel dos territórios na promoção de práticas alimentares promotoras de saúde (17).

Sabe-se que a implementação de políticas públicas eficazes para promover hábitos alimentares saudáveis exige a consideração das particularidades de cada contexto, dada a complexidade dos sistemas alimentares (18). O ambiente alimentar, especialmente em áreas urbanizadas, exerce grande influência nas escolhas alimentares dos indivíduos. Fatores como publicidade, informação e preços dos alimentos desempenham um papel crucial nesse processo, podendo tanto facilitar quanto dificultar a adoção de hábitos mais saudáveis (19). Por isso, é necessário criar estratégias que promovam ambientes alimentares mais saudáveis, considerando as especificidades de cada local.

A agricultura urbana e periurbana emerge, por exemplo, como uma estratégia para promover a segurança alimentar no Brasil. Ao envolver adolescentes e jovens, programas que fomentam esse tipo de produção agrícola podem fomentar hábitos alimentares mais saudáveis e a transição para sistemas alimentares mais sustentáveis (20).

A percepção positiva de corpo e saúde mostrou-se um fator relevante para a maior adesão às recomendações do Guia entre adolescentes, independentemente de características sociodemográficas. Essa associação evidencia a importância de abordar as questões alimentares e nutricionais durante a adolescência de forma integrada, considerando as mudanças físicas, psicológicas e sociais típicas dessa fase da vida (21). A insatisfação com o peso corporal e a distorção da imagem corporal são problemas comuns na adolescência e estão associados a comportamentos de risco à saúde, como práticas alimentares de risco para transtornos alimentares e baixa autoestima (22,23). Portanto, intervenções que promovam a aceitação do corpo e a autoestima podem ser eficazes na promoção de hábitos alimentares saudáveis e na prevenção de problemas de saúde relacionados à alimentação.

Nesse contexto, um estudo longitudinal com adolescentes noruegueses demonstrou que a preocupação com o corpo e o peso está associada a uma pior autoavaliação de saúde e autoestima, enquanto uma autopercepção positiva prediz um estado nutricional mais favorável (24). Essas evidências sugerem que promover a aceitação do corpo e a autoestima pode ser uma estratégia eficaz para melhorar práticas alimentares, além de prevenir a obesidade e os transtornos alimentares.

A autoavaliação positiva da saúde é necessária para promoção da alimentação saudável com adolescentes, pois associa-se, por exemplo, com o maior consumo de frutas e vegetais (25). Além disso, estudos anteriores demonstraram que indivíduos com melhor autopercepção de saúde tendem a apresentar comportamentos de vida mais saudáveis (26). No entanto, os resultados deste estudo sugerem uma relação paradoxal e mais complexa, pois além dos adolescentes com melhor autoavaliação de saúde apresentarem maior adesão às múltiplas recomendações do Guia, aqueles que autoavaliavam a saúde como ruim também se associaram a esse conjunto de práticas alimentares positivas.

Essa aparente contradição pode ser explicada pelo fato de que a percepção de vulnerabilidade à doença motiva, em alguns casos, a adoção de práticas mais saudáveis, como uma forma de tratamento de uma condição atual ou de prevenção de problemas de saúde futuros (27). Como destacado anteriormente, essa associação direta deve ser considerada com cuidado, pois as estimativas da RP no presente estudo também demonstram pouca precisão apesar da diferença estatística identificada. Porém, esse resultado que parece controverso deve ser considerado para o desenvolvimento de novos estudos quantitativos longitudinais e qualitativos para melhor compreensão desse fenômeno. Verifica-se nas ações práticas de cuidado alimentar e nutricional de adolescentes em serviços de saúde e educação que a vivência de questões de adoecimento por esse público ocasiona, algumas vezes, a mudança de práticas alimentares com a incorporação de orientações alimentares e nutricionais. 

O ambiente escolar é estratégico para a promoção da alimentação saudável entre adolescentes, porém as ações nesse sentido precisam ser qualificadas em escolas públicas e privadas. Destaca-se que não foram encontradas diferenças conforme o tipo de escola. Dado que o PNAE foi reestruturado em 2020 para se alinhar ao Guia e que os dados do artigo são de 2019, espera-se que tais mudanças poderão ser identificadas em resultados dos próximos estudos da PeNSE. Além disso, o alcance de mudanças mais efetivas nas práticas alimentares desse grupo etário parte da incorporação das diretrizes nacionais que orientem a oferta de alimentos saudáveis no contexto do PNAE, como as que consideram a cultura local e as recomendações do Guia, também nas cantinas escolares. Para isso, a participação da comunidade escolar (gestores, educadores, nutricionistas, pais, estudantes) e de diferentes setores (educação, saúde, comércio) é essencial para o sucesso dessas iniciativas (28).

Alinhadas ao Marco de Referência de Educação Alimentar e Nutricional (29) e à Lei nº 13.666/2018 (30), as ações que promovem a alimentação saudável nas escolas podem contribuir significativamente para a melhoria da adesão ao Guia entre adolescentes. O Decreto nº 11.821/2023 fortalece essa perspectiva, ao destacar a educação alimentar e nutricional, a doação e comercialização de alimentos e a comunicação mercadológica como eixos estratégicos para a promoção de ambientes alimentares saudáveis nas escolas (31).

Os resultados deste estudo possuem como fragilidade a utilização de marcadores de práticas alimentares que não foram desenvolvidos para medir de maneira precisa adesão ao Guia, principalmente ao considerar que seis passos preconizados por esse guia não puderam ser avaliados a partir das questões da PeNSE 2019. Porém, a representatividade dos dados para população de adolescentes escolares brasileiros e a robustez de pesquisa do IBGE ressaltam a importância das evidências construídas nesta pesquisa. Destaca-se que por tratar de um estudo transversal, existem algumas limitações inerentes a esse delineamento epidemiológico como: não estabelecer relações de causa e efeito, o não acompanhamento da população ao longo do tempo, e ser suscetível a variáveis de confusão que não foram coletadas. Tais fragilidades metodológicas podem ter gerado o resultado paradoxal da autopercepção de saúde discutido anteriormente. Por isso, os achados desta pesquisa devem ser verificados com atenção devido às limitações inerentes a esse tipo de estudo.

Por fim, verificou-se que a maior adesão a múltiplas recomendações do Guia entre adolescentes escolares do Brasil associou-se a fatores marcados por territórios menos urbanizados do país e com as percepções, principalmente, positivas sobre o corpo e a saúde. Ressalta-se que a associação paradoxal do desfecho com autoavaliação de saúde muito boa e ruim não devem ser desconsideradas e precisam ser exploradas em estudos futuros com esse público. Com isso, as intervenções para melhoria das práticas alimentares desse grupo etário devem basear-se na importância desses contextos territoriais e comportamentais para o desenvolvimento de ações mais efetivas.

## Data Availability

O banco de dados e os códigos de análise utilizados na pesquisa estão disponíveis em https://www.ibge.gov.br/estatisticas/downloads-estatisticas.html?caminho=pense/2009/microdados/.
